# Data analysis of striation spacing, lifetime, and crack length in crankshaft ductile cast iron under cyclic bending loading through high-cycle fatigue regime

**DOI:** 10.1016/j.dib.2022.108666

**Published:** 2022-10-12

**Authors:** Seyed Morteza Hosseini, Mohammad Azadi, Ahmad Ghasemi-Ghalebahman, Seyed Mohammad Jafari

**Affiliations:** a Faculty of Mechanical Engineering, Semnan University, Semnan, Iran; b Faculty of Mechanical and Energy Engineering, Shahid Beheshti University, Tehran, Iran

**Keywords:** Fatigue dataset, Ductile cast irons, Striation spacing, Lifetime, Crack length

## Abstract

In this dataset, experimental results of high-cycle bending fatigue testing on crankshaft ductile cast irons were presented both in raw and analyzed data. For this objective, EN-GJS-700-2 standard samples were cut and machined from the crankshaft of a gasoline engine. Then, stress-controlled rotary fatigue experiments were done on cast iron specimens under cyclic four-point bending loads in a fully-reversed condition (zero mean stress). These tests were considered under different cases of the loading rate and the applied stress, for both smooth and notched samples. The loading frequency was set to 12.5, 33.3, 58.3, and 100.0 Hz. The nominal stress was 226.6, 340.0, and 415.5 MPa in unnotched specimens. These values became 310.9, 513.6, and 642.4 MPa, respectively, when a notch was made on the specimens. After testing, field-emission scanning electron microscopy (FESEM) was utilized from the fracture surface of all samples to find the striation spacing and the crack length plus the fatigue lifetime. Obtained results from the sensitivity analysis illustrated that striation spacing was significantly affected by all three inputs of the loading frequency, the maximum stress, and the stress intensity factor. However, the loading frequency and the stress intensity factor had no effects on the fatigue lifetime and the crack length.

**Specifications Table**

**Table:** 

Subject	Engineering
Specific subject area	Engineering/ Mechanical Engineering/ Automotive Engineering
Type of data	Table, Image, and Figure
How the data were acquired	A rotary four-point bending fatigue testing device was utilized for cast iron standard samples under fully-reversed cyclic loadings. After the full failure (or fracture) of specimens, the lifetime was measured as cycles. Moreover, by the ImageJ software, the striation spacing and the crack length were calculated from the field-emission scanning electron microscopic (FESEM) images of the fracture surface. Then, experimental data were sensitively analyzed to find the effect of inputs including the loading frequency, the notch sample, and the stress level.
Data format	Raw data and Analyzed
Description of data collection	Cast iron standard smooth/notched samples were tested under fully-reversed four-point bending cyclic loads in the following conditions, Loading frequency: 12.5, 33.3, 58.3, and 100.0 Hz Nominal stress: 226.6, 340.0, and 415.5 MPa (in unnotched specimens) Maximum stress: 310.9, 513.6, and 642.4 MPa (in notched samples)
Data source location	Institution: Irankhodro Powertrain Company (IPCO) City/Town/Region: Tehran Country: Iran Latitude and longitude (and GPS coordinates, if possible) for collected samples/data: 35.70685873770743, 51.26710473353016
Data accessibility	Repository name: Mendeley Data Data identification number (permanent identifier, i.e., DOI number): 10.17632/xfgrxbst6p.1 Direct link to the dataset: https://data.mendeley.com/datasets/xfgrxbst6p/1

**Value of the Data**

• Crankshafts in automotive engines are under bending and torsional loads through the high-cycle fatigue regimes. The prediction of the fatigue lifetime of such a component is essential in the design process by engineers.
• Through such a complicated part in engines, different discontinuity issues could affect the fatigue lifetime. Therefore, knowing the notch influence could be helpful for designers.
• Fatigue testing is always time-consuming and has a high cost. Therefore, experimental data in this field of study could be valuable and helpful for researchers, especially for an industrial application of crankshafts.
• Standard fatigue samples were extracted from real crankshafts to consider the effect of manufacturing. This issue could be another important novelty besides the notch effect.
• Checking the influence of the loading rate or frequency and also the stress level could be considerable for crankshaft cast iron specimens.
• Scanning electron microscopic images could be utilized for the prediction of the fatigue lifetime based on striation spacing and the Paris law on the crack growth rate.
• Striation spacing on the fracture surface of fatigue test samples was used for the prediction of the fatigue lifetime.

## 1 Data Description

According to the dataset link (https://data.mendeley.com/datasets/xfgrxbst6p/1) in the Mendeley data, as the main experimental data, the field-emission scanning electron microscopy (FESEM) images were presented for the fracture surface of fatigue specimens. Moreover, a table was added for striation spacing and the fatigue lifetime related to samples, besides the loading frequency, the maximum stress, and the maximum stress at the notch.

**Fig. 1:**
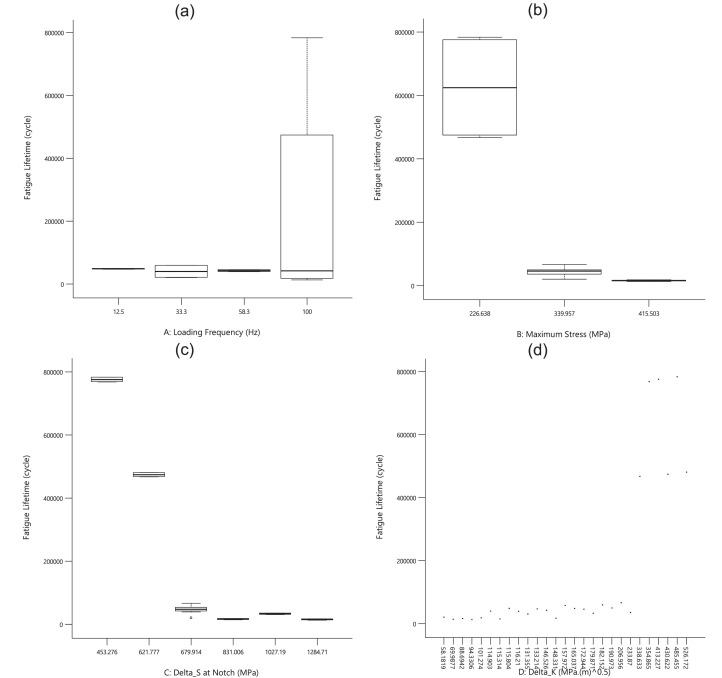
The scatter band of the fatigue lifetime for different inputs.

**Fig. 2:**
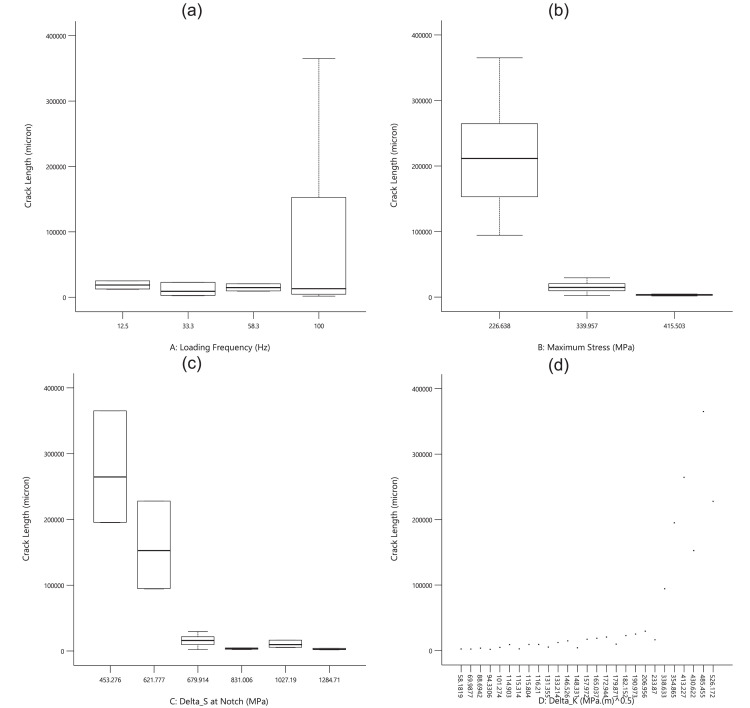
The scatter band of the crack length for different inputs.

**Fig. 3:**
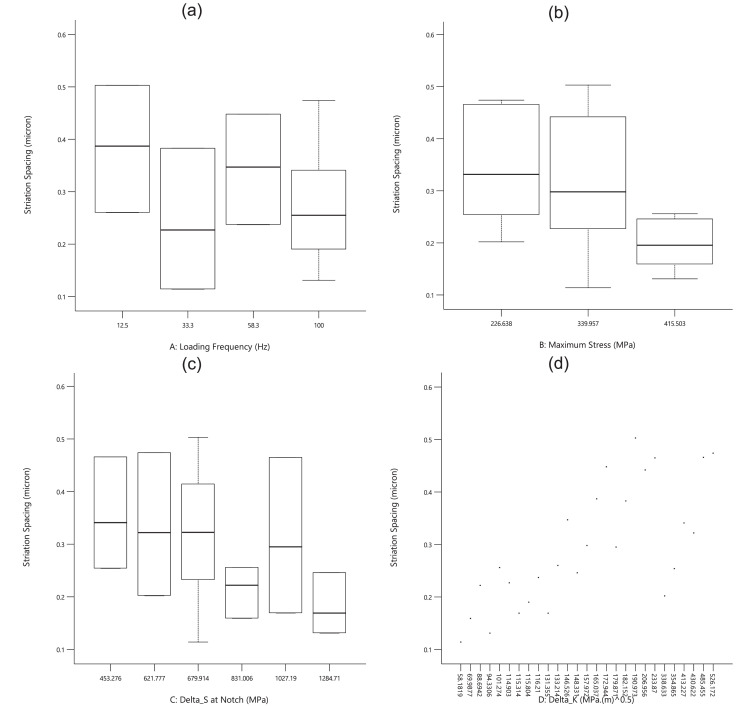
The scatter band of striation spacing for different inputs.

For the first experimental data, Fig. 1, Fig. 2, Fig. 3 show the averaged value and the standard deviation for the fatigue lifetime (*N_f,exp_*), the crack length (*a_exp_*), and striation spacing (*SS*) of cast iron samples, respectively. These outputs are based on different values of inputs including the loading frequency, the maximum stress (*S_max_*), the stress range (Δ*S*) at the notch, and the range of the stress intensity factor (Δ*K*). Notably, the maximum stress was calculated from the bending load (*S_max_*=*Mc*/*I; M*: moment, *c*: sample radius, *I*: the inertia moment). For the stress range (Δ*S*) at the notch, the mentioned formulation could be recalculated by considering the remained radius of the test sample with the notch depth of 0.5 mm.

The changing range of the stress intensity factor was as $\Delta K=\Delta S{\left(\pi a_{exp}\right)}^{0.5}$, where *a* is the crack length. On the other hand, the distance between the striations during the fatigue cycles can be equal to the crack length (the difference between the final crack length and the initial crack length). It should be mentioned that the initial crack length is assumed to be zero [1], [2]. The measurement of striation spacing at a specific value of the crack growth rate (d*a*/d*N*) could help the evaluation of both the damage accumulation and the crack extension [3], [4].

As an expected result, by increasing the stress level, the fatigue lifetime decreased on a logarithmic scale. Such a similar behavior could be seen for the stress range at the notch. The unexpected data for high loading frequency (100.0 Hz) would be investigated in the sensitivity analysis for a better understanding of the material behavior. Finally, the data for the stress intensity factor was scattered due to having different stress ranges and various crack lengths in all test samples. From the presented results in Fig. 2, a similar behavior could be seen for the crack length, compared to the fatigue lifetime of cast iron samples, versus different inputs. However, the changing trend for the stress intensity factor is continuously smoother for the crack length, compared to a sharp change for the fatigue lifetime.

Based on Fig. 3, all experimental output data had higher scatters versus the inputs. Therefore, no significant behavior could be predicted for striation spacing without any quantitative analysis. Furthermore, these data would be analyzed through sensitivity analysis in the next part. Just in the qualitative analysis, striation spacing decreased when the maximum stress was increased. This issue could be also reported in the literature by Aghareb Parast et al. [5]. Smaller areas for crack propagation could be seen on the fracture sample under a high-cycle fatigue regime (low stresses). For two other inputs of the loading frequency and the stress range at the notch, no obvious trend could be observed.

Until now, based on the presented results, only a qualitative analysis could be mentioned. Here, by a sensitivity analysis in the Design-Expert software, a quantitative analysis is represented. For this objective, the stress range at the notch was eliminated, where the stress intensity factor had a better rule on the outputs.

The linear regression model is presented in Table 1, Table 2, Table 3 for the fatigue lifetime, crack length, and striation spacing, respectively. These results are the summation of squares, the degree of freedom (df), the mean square, the F-value, and the P-value. Notably, a value lower than 0.05 for the P-value (equal to 95% of the confidence level) means the parameter is effective on the output and its influence is significant [6].

**Table 1:** The results of analyzed data for the fatigue lifetime of cast iron samples.

Source	Sum of Squares	df	Mean Square	F-value	P-value	Effectiveness
Regression Model	1.474E+12	3	4.913E+11	39.53	< 0.0001	significant
A: Loading Frequency	3.130E+10	1	3.130E+10	2.52	0.1262	not significant
B: Maximum Stress	7.593E+10	1	7.593E+10	6.11	0.0213	significant
D: Stress Intensity Factor	4.847E+10	1	4.847E+10	3.90	0.0604	not significant
Residual	2.859E+11	23	1.243E+10	-	-	-
R^2^	83.76%	-	-	-	-	-
Adjusted R^2^	81.64%	-	-	-	-	-
Predicated R^2^	75.94%	-	-	-	-	-

The mentioned linear regression models for all outputs versus the inputs are as follows,

(1) $$N_{f,exp}=\left(2.338+0.525A-1.856B+1.896D\right)\times 10^{5}$$

(2) $$a_{exp}=\left(1.118+0.035A-0.090B+1.455D\right)\times 10^{5}$$

(3) SS=0.437−0.083A+0.143B+0.300D

**Fig. 4:**
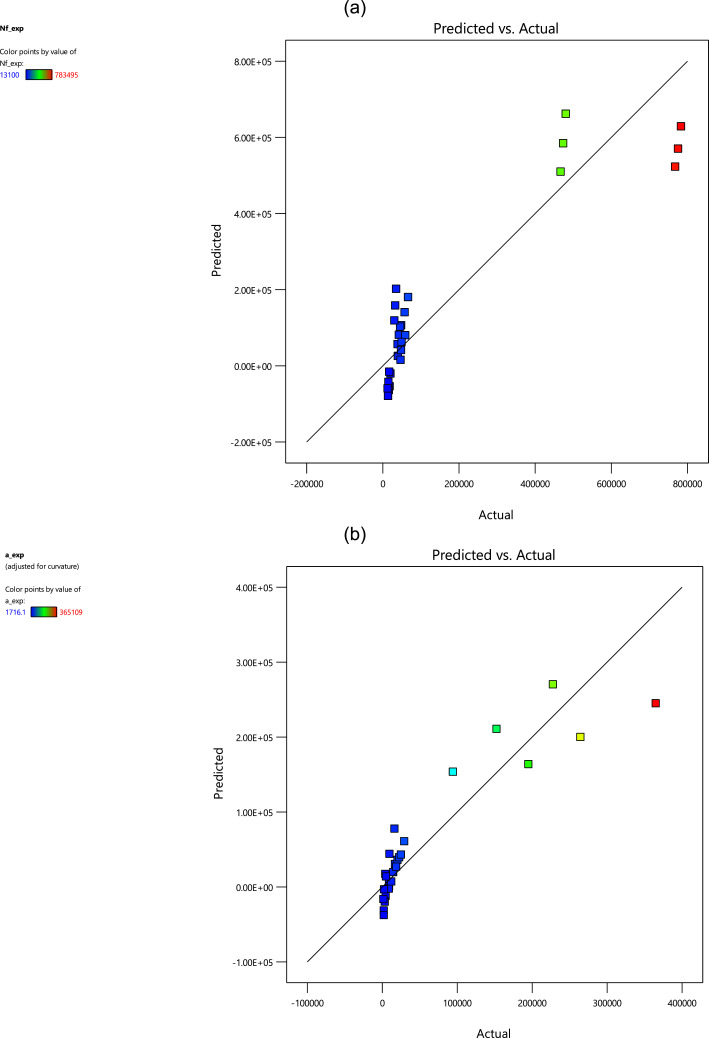

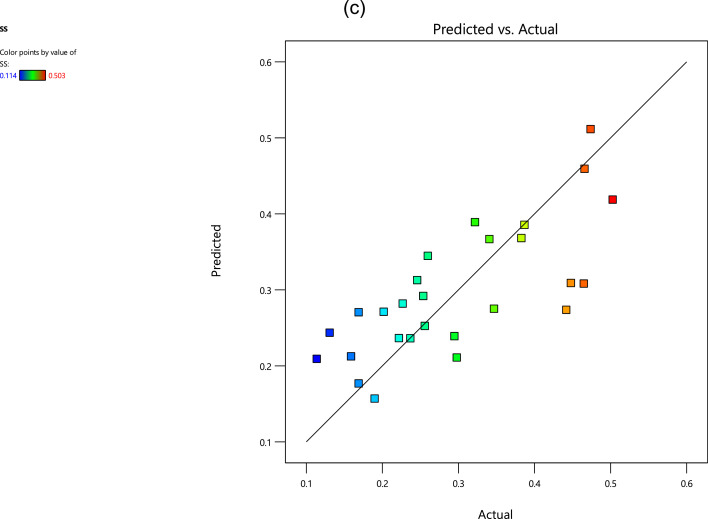
The scatter-band of the predicted and experimental values for (a) the fatigue lifetime, (b) the crack length, and (c) striation spacing.

Where *A* is the loading frequency, *B* is the maximum stress, and *D* is the stress intensity factor. Using such regression models for different outputs resulted in the scatter-band of the predicted value versus the experimental value. These scatter-bands could be seen in Fig. 4 for the fatigue lifetime, crack length, and striation spacing.

Based on the results in Fig. 4, it could be understood that the predicted and experimental data were scattered for striation spacing through the whole range. However, these data points (especially in the blue color) were gathered in one area for the fatigue lifetime and the crack length in Figs. 4(a) and 4(b), respectively.

**Fig. 5:**
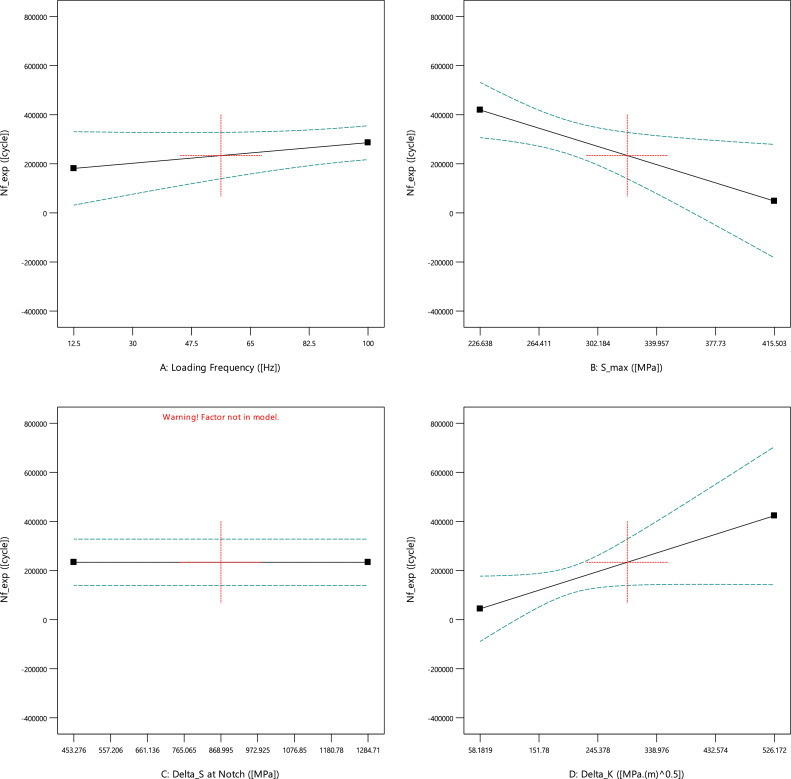
The effect of input parameters on the fatigue lifetime of cast iron samples.

**Fig. 6:**
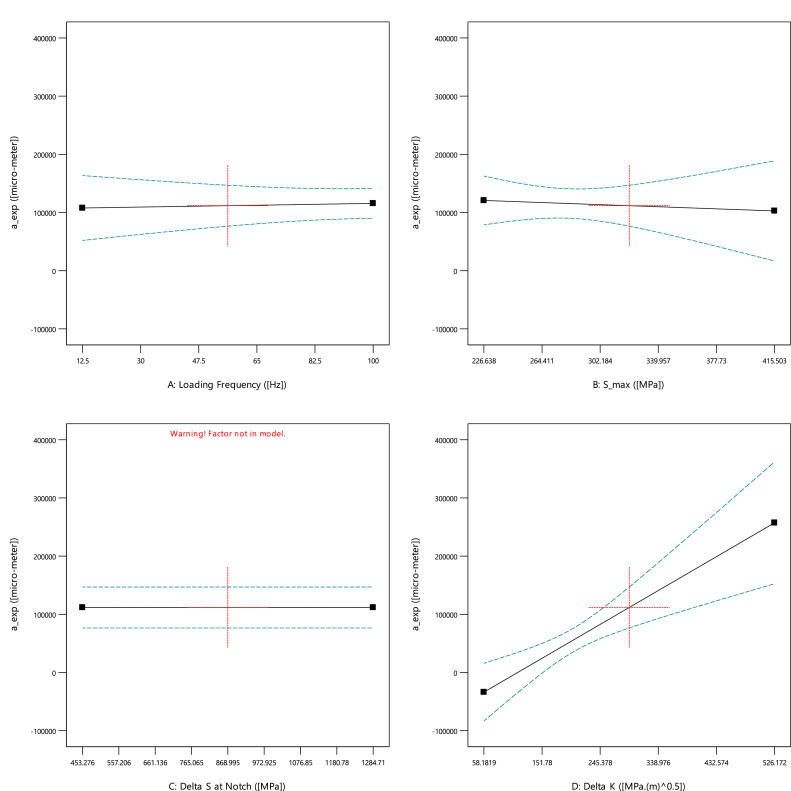
The effect of input parameters on the crack length of cast iron samples.

**Fig. 7:**
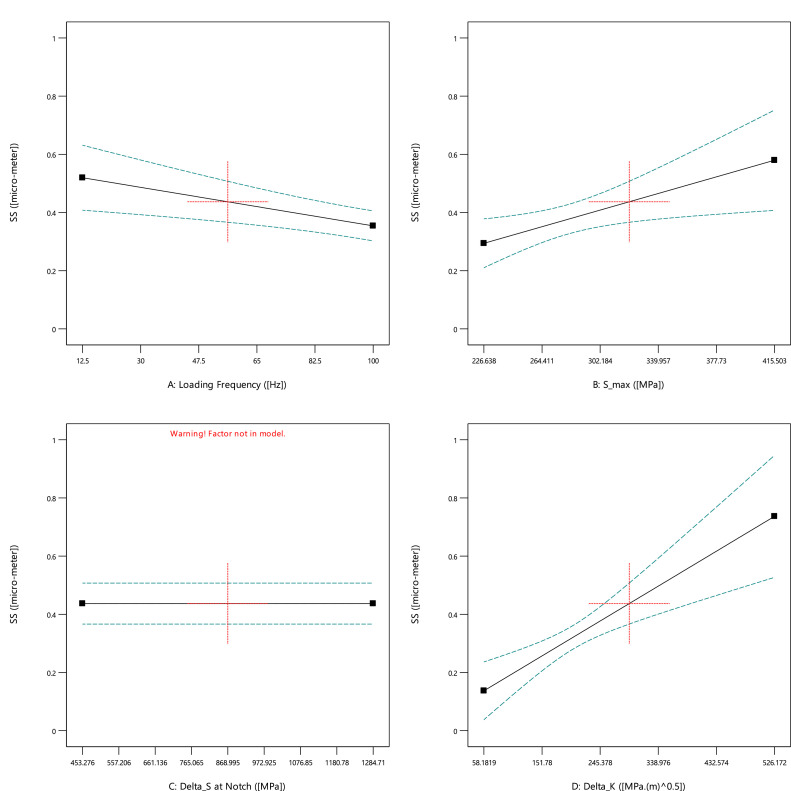
The effect of input parameters on striation spacing of cast iron samples.

The changing trend of each output versus the inputs could be seen in Fig. 5, Fig. 6, Fig. 7. In these cases, the stress range at the notch was not modeled and predicted and therefore, only a horizontal line could be seen.

Based on Fig. 5, the fatigue lifetime increased gradually with the loading frequency. By increasing the maximum stress, as mentioned before, the fatigue lifetime decreased sharply. This trend behavior could be also reversely reported for the stress intensity factor. Notably, Table 1 shows the maximum stress, as the only significant parameter on the objective of the fatigue lifetime. Other parameters of the loading frequency and the stress intensity factor were not effective. It should be noted that the P-value of the stress intensity factor was 0.0604, which demonstrated a non-significant factor but near the value of 0.05 (the criterion for effectiveness).

According to Fig. 6, the crack length was not affected by the loading frequency and the maximum stress (also shown in Table 2). However, the stress intensity factor had a significant influence on the crack length of cast iron samples. The crack length enhanced sharply when the stress intensity factor increased.

**Table 2:** The results of analyzed data for the crack length of cast iron samples.

Source	Sum of Squares	df	Mean Square	F-value	P-value	Effectiveness
Regression Model	2.020E+11	3	6.734E+10	39.01	< 0.0001	significant
A: Loading Frequency	1.771E+08	1	1.771E+08	0.10	0.7516	not significant
B: Maximum Stress	1.786E+08	1	1.786E+08	0.10	0.7506	not significant
D: Stress Intensity Factor	2.854E+10	1	2.854E+10	16.54	0.0005	significant
Residual	3.970E+10	23	1.726E+09	-	-	-
R^2^	83.58%	-	-	-	-	-
Adjusted R^2^	81.43%	-	-	-	-	-
Predicated R^2^	74.82%	-	-	-	-	-

Based on Fig. 7 and Table 3, all inputs had significant effects on striation spacing. When the loading frequency increased, striation spacing decreased. Moreover, while the maximum stress and also stress intensity factor enhanced, striation spacing increased, too. This result (the maximum stress versus striation spacing) was reversed in Fig. 3(b). The reason could be due to the low value of R^2^ in the regression analysis. This issue could be proved by observing a high value for the standard deviation at 340.0 MPa in Fig. 3(b). As another reason from Table 3, the F-value for the maximum stress was 6.50, lower than all other values for other parameters. It means that the least effective parameter on striation spacing was the maximum stress. However, Ahmed et al. [7] reported that striation spacing increased when the stress decreased for commercially pure titanium. They illustrated that the threshold crack length was 2.5, 1.9, and 1.4 mm, where striation spacing started to increase at 175, 200, and 227 MPa, respectively [7]. The related minimum value for striation spacing was also reported as 0.45, 0.36, and 0.24 µm, for the mentioned applied stress [7].

**Table 3:** The results of analyzed data for striation spacing of cast iron samples.

Source	Sum of Squares	df	Mean Square	F-value	P-value	Effectiveness
Regression Model	0.191	3	0.064	9.19	0.0004	significant
A: Loading Frequency	0.078	1	0.078	11.24	0.0028	significant
B: Maximum Stress	0.045	1	0.045	6.50	0.0180	significant
D: Stress Intensity Factor	0.121	1	0.121	17.52	0.0004	significant
Residual	0.159	23		-	-	-
R^2^	54.51%	-	-	-	-	-
Adjusted R^2^	48.57%	-	-	-	-	-
Predicated R^2^	41.24%	-	-	-	-	-

As an important note, Ripplinger et al. [8] found that by increasing the pearlite concentration and thereby the static and fatigue strength in the near-surface region at a notch, a significant increase of the fatigue strength was achieved. Therefore, it could be suggested that the effect of graphite nodules and the amount of pearlite on the experimental results are considered for future research.

**Fig. 8:**
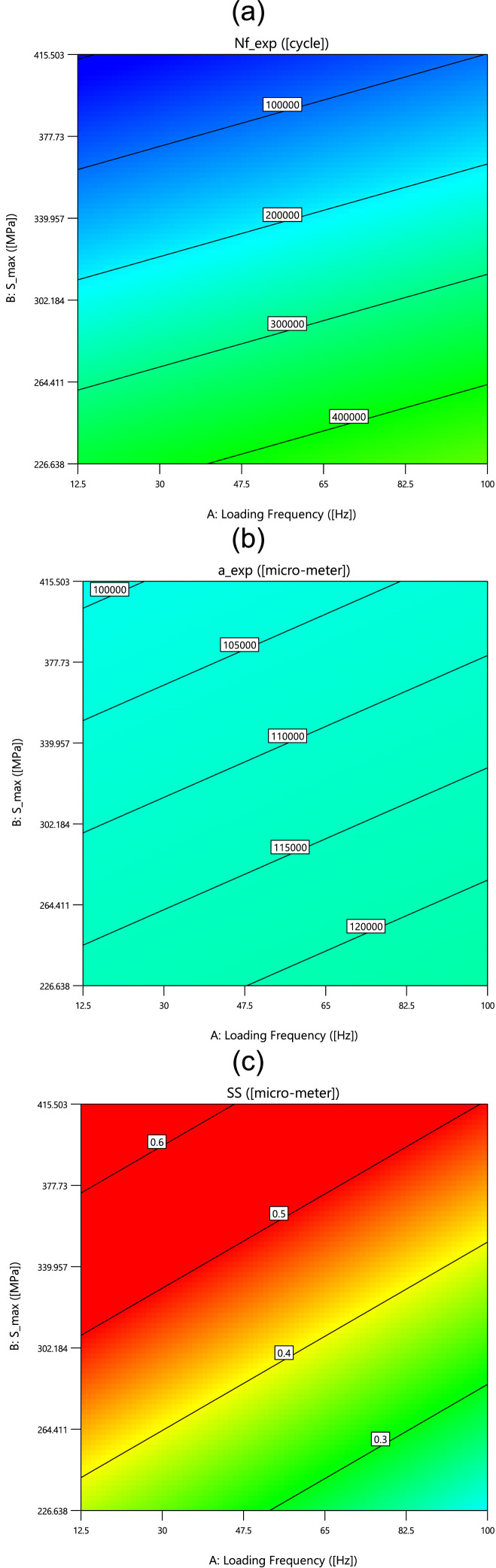
The contour plots of outputs versus the loading frequency and the maximum stress.

**Fig. 9:**
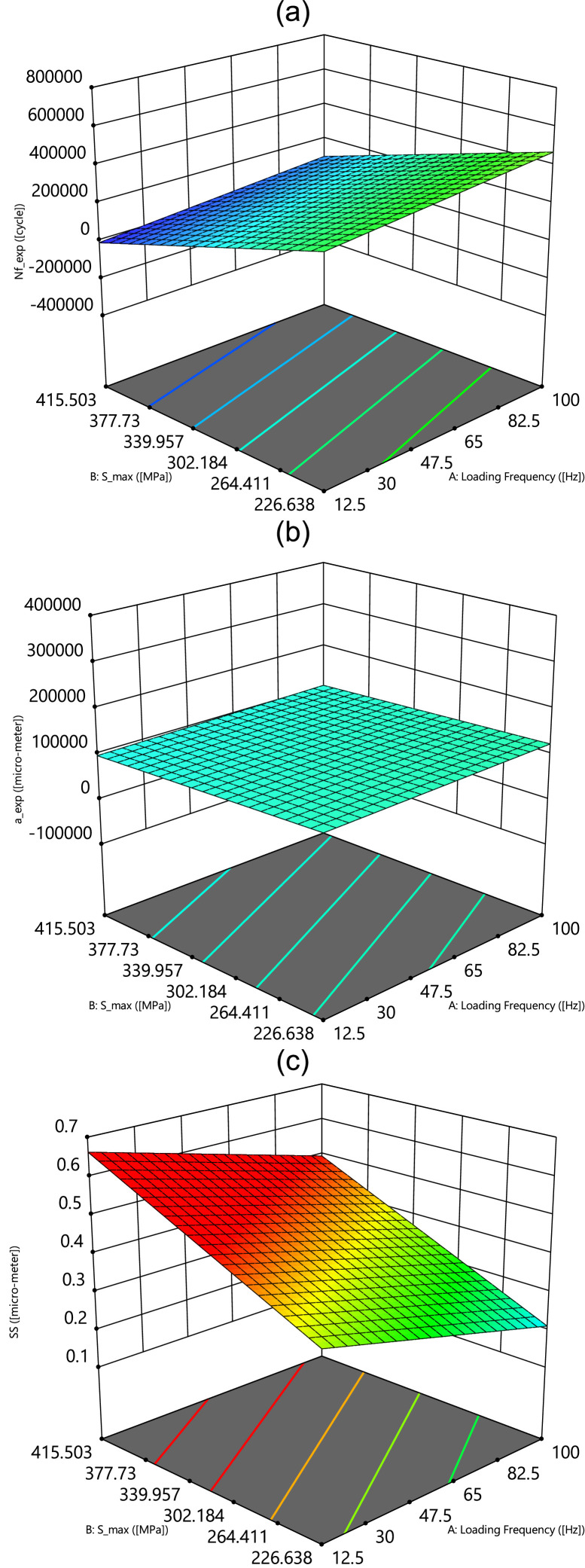
The surface plots of outputs versus the loading frequency and the maximum stress.

Finally, as the last results, the contour plots and the surface plots of three outputs versus the loading frequency and the maximum stress are mentioned in Fig. 8, Fig. 9, respectively. From these results, having no influences from inputs on the crack length is clear with one color contour and smooth surface.

## 2 Materials and Experiments

**Fig. 10:**
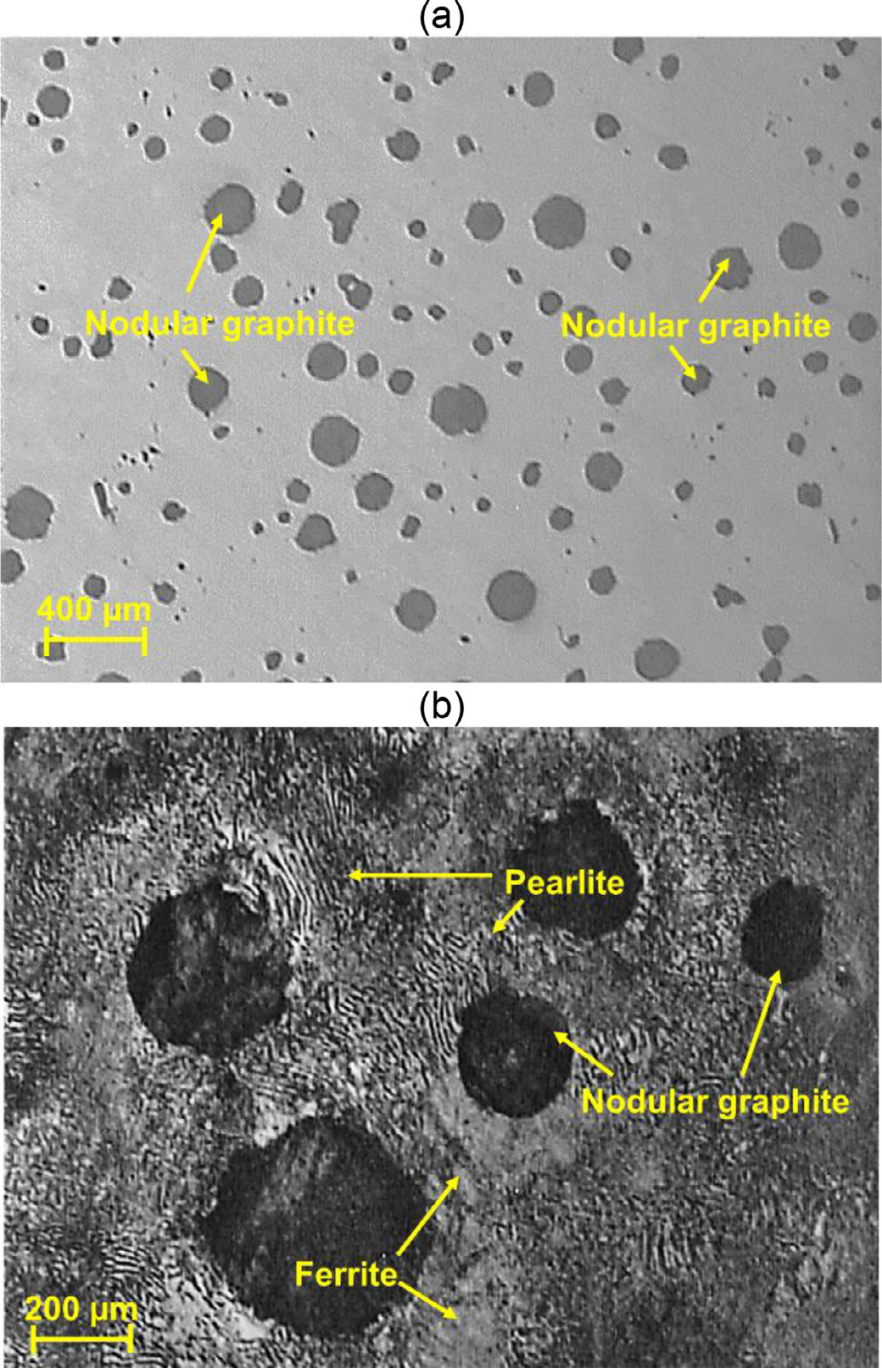
The DCI microstructure of EN-GJS-700-2: (a) without and (b) with an etchant of 2% Nital.

The investigated material was a ductile cast iron (DCI) with the application of crankshafts in gasoline engines. The chemical composition of the DCI (EN-GJS-700-2) is given in Table 4. The mechanical properties of DCI are directly related to its microstructure. The microstructure of this cast iron includes the nodular graphite in the matrix of ferrite and pearlite. Figs. 10(a) and 10(b) show the microstructure of DCI (EN-GJS-700-2), with and without the use of an etchant solution (2% Nital [9]), respectively. While in Fig. 10(a), only spherical graphites are visible, Fig. 10(b) also illustrates pearlite and ferrite phases. The observed microstructure contained the typical phases of EN-GJS-700-2 including pearlite-ferrite phases and spherical graphites, as also Hosseini et al. [9], Asi [10], and Khameneh and Azadi [11] had stated. The mechanical properties of crankshaft materials are also given in Table 5, using the ISO-6892 standard [12].

**Table 4:** The chemical composition of the DCI: EN-GJS-700-2 (% wt.).

C	Si	Mn	P	S	Cu	Ni	Sn	Cr	Al	Ti	V	W	Co	Nb
3.50	2.18	0.45	0.01	0.01	0.48	0.02	0.05	0.03	0.02	0.04	>0.01	>0.01	>0.01	>0.01

**Table 5:** The mechanical properties of the DCI: EN-GJS-700-2.

Mechanical Properties	Unit	Mean Value	Standard Deviation
Tensile strength	MPa	466.83	13.59
Yield strength	MPa	293.65	21.58
Elastic module	GPa	117.37	350.42
Elongation	%	9.44	0.14

It should be noted that the crack propagation resistance of DCIs depends on the loading condition, the chemical composition, the matrix microstructure, and the graphite element morphology [13], [14], [15], [16], [17]. Cavallini et al. [18] investigated the effect of micro-mechanisms on the resistance of fatigue cracks in DCIs at different stress levels. They examined the crack paths by scanning the crack path profile and analyzing the fracture surfaces with a scanning electron microscope. Their results illustrated that the micro-mechanisms affected the fatigue crack propagation resistance of DCIs. Fonte et al. [19] performed failure analysis on a crankshaft of a boxer diesel engine and their results demonstrated that the crack surface created in the fillet area of the crankshaft pin due to the stress concentration or loading conditions. Di Cocco and Iacoviello [20] investigated the effect of the microstructure on damage micro-mechanisms in the field of fatigue cracks due to overload. They concluded that the increase in the damage level was evidence of the influence of ferritic and ferritic-pearlite DCIs. Whereas in the pearlite DCI, there was no significant transition between fatigue and failure due to overloading. In a study by Bellini et al. [21], they studied the damage micro-mechanisms in the pearlitic DCI, using scanning electron microscopy. During in situ uniaxial tensile testing on micro-tensile specimens under strain-controlled loading conditions, they detected multiple damage-conducting micro-mechanisms. Di Cocco and Iacoviello [22] found the influence of the graphite nodules morphology on the mechanical properties of pearlitic DCIs in static, quasi-static, and cyclic loading conditions. They depicted that matrix-nodule debonding was the most important mechanism in failures. In other words, the predominant micro-mechanism on the fracture surface of the fatigue test specimens was the debonding process of nodules from the matrix.

**Fig. 11:**
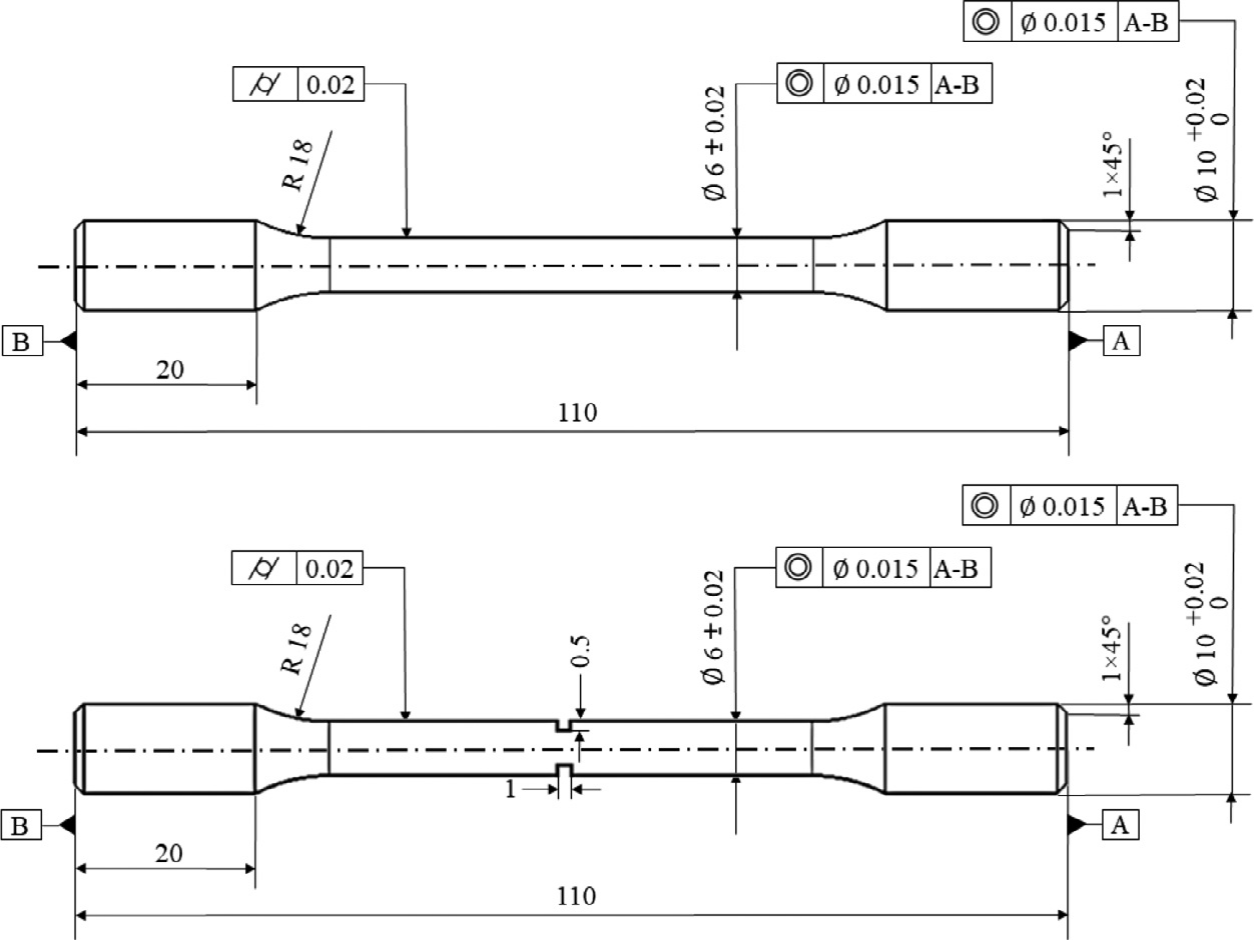
The dimension (in mm) of standard fatigue specimens for smooth and notched cases.

Some crankshafts of a gasoline engine were used to extract and machine the fatigue testing sample from the web area. This issue could be considered to find the influence of the fabrication method on the performance of real automotive components. The dimension of standard samples for fatigue testing is demonstrated in Fig. 11. More details for manufacturing the specimens could be found in the literature [9].

A module for acoustic emission sensors was also used during fatigue testing, which is not important here and the results will be presented in the near future. The objective was to acquire acoustic emission signals for another prediction approach of the fatigue lifetime. On the other hand, the purpose of considering a notch in some specimens was to control the location of the fracture to identify the initiation of the fatigue crack in the acoustic emission signals. Therefore, the notch was a circumferential notch with a U-shaped section on the surface of some specimens with a depth of 0.5 mm and a thickness of 1 mm.

**Fig. 12:**
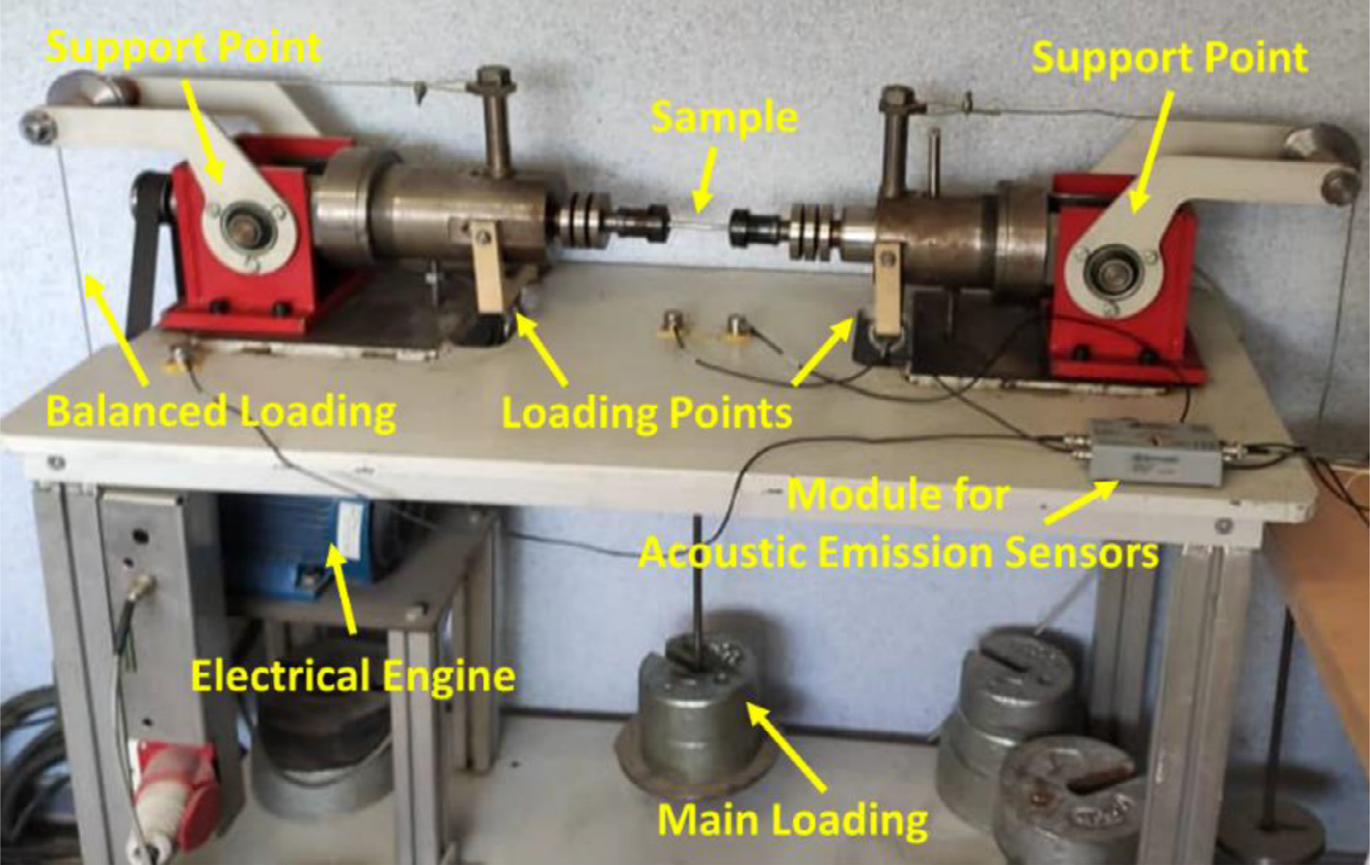
The device for force-controlled four-point rotary bending high-cycle fatigue testing.

Under the loading rate of 100.0 Hz (6000 rpm) and lower values, besides R=-1 (zero mean stress), four-point force-controlled rotary fatigue experiments (Fig. 12) were done for fully-reversed cyclic bending loads. Notably, this loading frequency was selected due to the rated power conditions in gasoline engines [11]. These tests were performed through the high-cycle fatigue (HCF) regime at room temperature based on the ISO-1143 standard [23]. Finally, the loading frequency was considered 12.5, 33.3, 58.3, and 100.0 Hz for the nominal stress of 226.6, 340.0, and 415.5 MPa (in unnotched specimens) and the maximum stress of 310.9, 513.6, and 642.4 MPa (in notched samples).

For the sensitivity analysis, all inputs and outputs are illustrated in Tables 6 and 7. Notably, all input parameters are numeric and continuous. Then, the linear regression analysis was performed by the Design-Expert software. Then, the input influences were investigated on the outputs.

**Table 6:** The inputs for the sensitivity analysis of cast iron samples.

Factor	Name	Units	Minimum	Maximum	Coded Low	Coded High	Mean	Std. Dev.
A	Loading Frequency	Hz	12.50	100.00	-1 ↔ 12.50	+1 ↔ 100.00	78.23	33.25
B	Maximum Stress	MPa	226.64	415.50	-1 ↔ 226.64	+1 ↔ 415.50	331.56	64.86
C	Stress Range at Notch	MPa	453.28	1284.71	-1 ↔ 453.28	+1 ↔ 1284.71	770.85	237.67
D	Stress Intensity Factor	MPa(m)^0.5^	58.18	526.17	-1 ↔ 58.18	+1 ↔ 526.17	202.70	131.38

**Table 7:** The outputs for the sensitivity analysis of cast iron samples.

Response	Name	Units	Observations	Minimum	Maximum	Mean	Std. Dev.	Ratio
Striation Spacing	*SS*	µm	27	0.114	0.503	0.297	0.116	4.41
Fatigue Lifetime	*N_f,exp_*	cycle	27	13100.000	783495.000	1.670E+05	2.602E+05	59.81
Crack Length	*a_exp_*	µm	27	1716.100	365109.000	57086.410	96422.900	212.75

It should be noted that one input parameter is the stress intensity factor, which is generally the multiplier of the stress and the crack length ($\Delta K=\Delta S{\left(\pi a_{exp}\right)}^{0.5}$). In other words, the interaction effect of two inputs including the stress and the crack length was considered in the regression model (for the sensitivity analysis). For physical meanings, the regression analysis was not exactly linear by considering the stress intensity factor and it was only mathematically linear.

As another note, according to the previous research [1], [2], [3], [4], the shape of the crack to calculate the stress intensity factor was assumed to be a circumferential two-dimensional crack. The value of striation spacing, related to the crack length, was measured from the microscopic 2D-images, in this study.

Moreover, in other similar studies [24], [25], [26], [27], the fatigue crack length was measured for ductile cast irons, which could be compared and analyzed with the results of the present study. The crack length was between 1.7 and 29.5 mm in the present work (neglecting Specimen No. “DIC_3” and “DIC_9”), compared to 0.1-9.1 in the literature [24], [25]. In this comparison, the results of samples are considered unless Specimen No. “DIC_3” and “DCI_9”. The related crack length was 274.9 ± 69.8 and 158.3 ± 54.7 mm, respectively. The reason is due to the fatigue lifetime, which was the highest value in these specific specimens among all specimens. The fatigue lifetime in Specimen No. “DIC_3” and “DIC_9” was 775,798 ± 6,285 and 474,152 ± 5,513 cycles, respectively. These values are in the order of 10 times higher than other fatigue lifetimes.

Connors [28] and other researchers [29] established a linear relationship between striation spacing and the crack length for a specific crack length range. On the other hand, above a specific crack length, this relationship becomes nonlinear. Considering that issue, the average values of striation spacing were between 0.1 and 0.5 μm, and therefore, the linear extension of the plastic zone is small compared to the significant dimensions of the body, especially the crack length [30], [31].

**Fig. 13:**
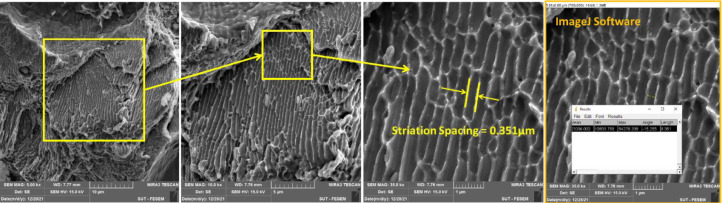
The measurement process of striation spacing in the ImageJ software: An example for Specimen No. “DCI_3(2)” with one data for measuring.

The file name of scanning electron microscopic images could be found in the appendix, based on this table and the mentioned sample number. An example for measuring striation spacing on the fractured sample could be observed in Fig. 13, using the ImageJ software. It should be noted that the data of 0.351 µm for striation spacing is just a sample for Specimen No. “DCI_3(2)”. Since the data for striation spacing are the averaged values of different measuring.

## Ethics Statements

Generally, it is not applicable to this analyzed dataset. Moreover, these data are obtained from experimental investigations without any relations to human and animal issues.

## CRediT Author Statement

**Mohammad Azadi:** Conceptualization, Methodology, Investigation, Validation, Writing – original draft preparation, Writing – reviewi & editing, Supervision. **Seyed Morteza Hosseini:** Methodology, Investigation, Validation, Writing – review & editing, Data curation, Software, Visualization; **Ahmad Ghasemi-Ghalebahman:** Conceptualization, Methodology, Investigation, Supervision; **Seyed Mohammad Jafari:** Methodology, Investigation, Supervision, Data curation, Software.

## Declaration of Competing Interest

The authors declare that there are no known competing financial interests or personal relationships for this work.

## Data Availability

Scanning electron microscopic images of fractured cast iron samples under cyclic loading (Original data) (Mendeley Data).
